# Human Meibum and Tear Film Derived (O-Acyl)-Omega-Hydroxy Fatty Acids as Biomarkers of Tear Film Dynamics in Meibomian Gland Dysfunction and Dry Eye Disease

**DOI:** 10.1167/iovs.62.9.13

**Published:** 2021-07-08

**Authors:** Safal Khanal, Yuqiang Bai, William Ngo, Kelly K. Nichols, Landon Wilson, Stephen Barnes, Jason J. Nichols

**Affiliations:** 1 Department of Optometry and Vision Science, School of Optometry, University of Alabama at Birmingham, Birmingham, Alabama, United States; 2 Centre for Ocular Research & Education, School of Optometry & Vision Science, University of Waterloo, Waterloo, Ontario, Canada; 3 Centre for Eye and Vision Research, 17W Hong Kong Science Park, Hong Kong; 4 Department of Pharmacology and Toxicology, School of Medicine, University of Alabama at Birmingham, Birmingham, Alabama, United States; 5 Targeted Metabolomics and Proteomics Laboratory, University of Alabama at Birmingham, Birmingham, Alabama, United States

**Keywords:** tear film, (O-Acyl)-omega-hydroxy fatty acids, lipids, meibum, precorneal tear film, tear film lipid layer, meibomian gland, dry eye disease

## Abstract

**Purpose:**

To investigate the association between precorneal tear film (PCTF)– and meibum-derived (O-Acyl)-omega-hydroxy fatty acids (OAHFAs) and PCTF thinning in meibomian gland health and dysfunction.

**Methods:**

Of 195 eligible subjects (18–84 years, 62.6% female), 178 and 170 subjects provided both PCTF optical coherence tomography (OCT) imaging and mass spectrometry data for tears (n = 178) and meibum (n = 170). The PCTF thinning rate was measured in the right eye using an ultra-high-resolution, custom-built OCT. Tear and meibum samples from the right eye were infused into the SCIEX 5600 TripleTOF mass spectrometer in the negative ion mode. Intensities (*m/z*) of preidentified OAHFAs were measured with Analyst 1.7TF and LipidView 1.3 (SCIEX). Principal component (PC) analyses and Spearman's correlations (*ρ*) were performed to evaluate the association between OAHFAs and PCTF thinning rates.

**Results:**

In meibum and tear samples, 76 and 78 unique OAHFAs were detected, respectively. The first PC scores of the meibum-derived OAHFAs had statistically significant correlations with PCTF thinning rates (*ρ* = 0.18, *P* = 0.016). Among 10 OAHFAs with the highest first PC loadings, six OAHFAs had negative correlations with PCTF thinning rate (18:2/16:2, *ρ* = −0.19, *P* = 0.01; 18:2/30:1, *ρ* = −0.21, *P* = 0.008; 18:1/28:1, *ρ* = −0.22, *P* = 0.004; 18:1/30:1, *ρ* = −0.22, *P* = 0.005; 18:1/25:0, *ρ* = 0.22, *P* = 0 .006; and 18:1/26:1, *ρ* = −0.22, *P* = 0.006), while one OAHFA had a positive correlation with PCTF thinning rate (18:2/18:1, *ρ* = 0.48, *P* = 0.006). Tear film-derived OAHFAs had no association with the PCTF thinning rate.

**Conclusions:**

Several human meibum-derived OAHFAs showed significant associations with PCTF thinning, suggesting that these OAHFAs could be implicated in the mechanism underlying the stabilization and thinning of the PCTF. The tear-film derived OAHFAs were, however, independent of the rate of PCTF thinning.

A thin film (∼2 to 5 µm)^1^^–^^3^ of semiviscous tear fluid covers the ocular surface. Shortly after a blink, the human precorneal tear film (PCTF) thins and breaks up abruptly (∼seconds), but again spreads over the ocular surface and restores its thickness following redistribution by a subsequent blink.^4^ The PCTF thinning is primarily due to evaporative loss of aqueous from the PCTF rather than other mechanisms, such as tangential or radial fluid flow or gravity.^4^^–^^6^ When aqueous evaporation is excessive, a cascade of events is initiated, resulting in tear hyperosmolarity and inflammatory damage to the ocular surface.^7^^–^^9^ According to the Tear Film Ocular Surface Society (TFOS) Dry Eye Workshop (DEWS II), evaporation-induced tear film instability is a hallmark mechanism that triggers the vicious cycle of dry eye disease (DED).^10^^,^^11^

Overlying the mucoaqueous phase of the human tear film is a thin layer of lipids (mean 42 nm, range, 15 to 157 nm)^12^ that forms a barrier against evaporative aqueous loss,^9^^,^^13^^–^^16^ and renders elasticity to the air-tear surface, providing resistance to stretching and thinning deformations and conferring mechanical stability to the tear film.^17^^–^^19^ This tear film lipid layer (TFLL) consists of a complex mixture of non-polar (e.g., wax esters, cholesteryl esters) and polar lipids (e.g., phospholipids, (O-Acyl)-omega-hydroxy fatty acids [OAHFAs]),^19^^–^^21^ which are predominantly derived from meibum secreted by the meibomian glands.^22^^,^^23^ Therefore the ability of the TFLL to retard evaporation and protect against aqueous loss depends to a large extent on meibomian gland health. Qualitative or quantitative alterations in meibum resulting from meibomian gland dysfunction (MGD) may affect PCTF structure, function, and dynamics and may produce deficiencies in lipid constituents of the TFLL, inhibiting its ability to spread the nonpolar lipids evenly over the mucoaqueous phase.^10^^,^^24^ Indeed, MGD is recognized as the leading cause of evaporative DED.^10^^,^^25^ Studies have shown that the expression of meibum increases PCTF stability and TFLL thickness^26^ and decreases the rate of tear evaporation in healthy subjects and those with coexisting DED and MGD.^27^ Conversely, the PCTF has been found to be unstable and the rate of tear evaporation increased in subjects with absent or less confluent lipid layer.^14^ Clinical and population-based studies also indicate a high rate of comorbidity of MGD and DED,^24^^,^^28^^–^^32^ suggesting a strong association between meibomian gland health and PCTF organizational characteristics.

Although changes in PCTF dynamics are better established in DED, the molecular mechanisms or alterations underlying these physiological changes have yet to be fully understood. This is partly due to the complex relationship between the TFLL thickness and PCTF thinning, because studies have shown at best a modest negative association between these two parameters, with some even reporting a positive association between thicker TFLL and clinical signs of DED.^6^^,^^33^ These findings have stimulated the hypothesis that the TFLL interacts with other tear film constituents (e.g., mucins, proteins, and salts) to function as an antievaporative barrier.^19^ However, factors other than TFLL structure (thickness) such as the biochemical composition are becoming recognized as being critical.^34^^,^^35^ A growing body of literature now suggests that the TFLL composition and the biophysical properties of its constituents may control the rate of PCTF thinning or evaporation of tears from the ocular surface.^21^^,^^36^^–^^41^

Of all tear film lipids, OAHFAs, a subclass of amphiphilic anionogenic lipids more recently discovered in human meibum,^42^ are a strong lipid biomarker candidate involved in maintaining the TFLL^41^^,^^43^ and imparting a direct evaporation-resistant effect to stabilize the PCTF.^44^^,^^45^ Several studies have demonstrated the presence of OAHFAs in human meibum and tear film^46^^–^^50^ and suggested their potential role in PCTF thinning and stabilization.^43^^,^^50^^,^^51^ In mice, deficiency of OAHFA-producing ω-hydroxylase Cyp4f39 fatty acid led to dry eyes and MGD.^43^ Cyp4f39-deficient mice also showed decreases in specific OAHFAs and their derivatives, suggesting that this reduction likely caused DED and MGD, although findings were somewhat inconclusive because of limited sample size, no changes in other major OAHFAs, and confounding observations such as corneal epithelial damage, which may have instead contributed to the observed dry eye.^43^ In humans, OAHFAs were found to decrease with increasing severity of DED^49^ and increase after eyelid warming treatment in MGD.^51^

On the basis of these findings, it is hypothesized that OAHFAs are implicated in the mechanism underlying the destabilization and thinning (evaporation) of the PCTF. The present study was designed to test this hypothesis by investigating the association between the abundance of meibum- and tear film–derived OAHFAs with PCTF structural characteristics.

## Materials and Methods

Ethics approval for this study was obtained from the Institutional Review Board of the University of Alabama at Birmingham. Each subject gave informed consent in writing before participation in the study, and all study procedures adhered to the tenets of the Declaration of Helsinki.

### Nomenclature

**Figure 1.**
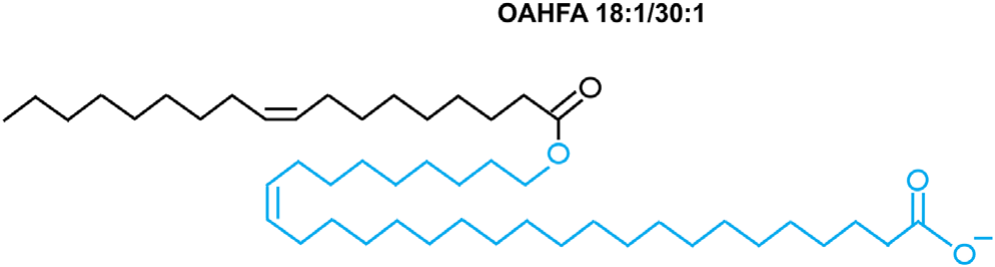
Schematic diagram of the structure of OAHFA18:1/30:1, which contains 18 carbons in the fatty acid chain (*black*) and 30 carbons in the hydroxyl fatty acid chain (*blue*), with one double bond in each chain.

OAHFAs are large molecular weight molecules of a fatty acid esterified with an omega-hydroxy fatty acid. The number of bonds in the aliphatic chain determines their degree of saturation. Saturated OAHFAs contain no double bonds, whereas unsaturated OAHFAs contain one or more double bonds.^52^ Standard nomenclature for OAHFAs is used in this study^53^^,^^54^; OAHFA X1:Y1/X2:Y2, where X1 and Y1 denote the number of carbons and double bonds in the fatty acid component, respectively, and X2 and Y2 denote the number of carbons and double bonds in the omega-hydroxy fatty acid component, respectively. Figure 1 shows the structure of an example OAHFA 18:1/30:1.

### Human Subject Recruitment

To determine the eligibility of subjects for participation in the study, a prescreening was carried out among subjects who had previously provided consent to be contacted for future studies as part of the University of Alabama at Birmingham Clinical Eye Research Facility IRB approved recruitment databases and procedures. Subjects were included if they were non-contact lens wearers and 18 years of age or older. Subjects receiving eye care for acute ocular diseases (except MGD or DED), currently using topical ophthalmic lubricants or medications, having health or ocular conditions that could impact meibomian gland function or tear film parameters, and currently participating in any other intervention-based clinical research study, were excluded. Based on the prescreening, a total of 222 subjects consented to participate in the study.

### Clinical Protocol

All subjects attended a single study visit in the Clinical Eye Research Facility at the University of Alabama at Birmingham. After a detailed ocular and systemic history, subjects completed the Ocular Surface Disease Index (OSDI) questionnaire^55^ and underwent a comprehensive examination of the adnexa and ocular surface including tear film imaging.^56^ The expressibility and quality of meibum from the meibomian gland orifices were examined by applying firm digital pressure to the lower eyelid. Each gland was graded on a scale of 0 to 3, with 0 representing clear fluid, 1 representing cloudy liquid, 2 representing cloudy with debris, and 3 representing toothpaste-like thick secretion.^57^ The sum of the grades from the central eight glands provided the clinical meibum grade score. Based on the OSDI and meibum grade score, each subject was classified into one of the four disease classification groups, adapted from the TFOS International Workshop on MGD guidelines: Normal (OSDI < 13 and meibum grade < 10), MGD (OSDI ≥ 13 and meibum grade ≥ 10), Asymptomatic MGD (OSDI < 13 and meibum grade ≥ 10), and Mixed (OSDI ≥ 13 and meibum grade < 10).^58^

Tear film imaging techniques are described elsewhere.^56^ Briefly, cross-sectional OCT images of the tear film were captured from the right eye of each subject in a dimly illuminated room at a controlled temperature (23°–25°C) and humidity (30%–50%). During imaging, subjects were instructed to blink three times and then keep their eyes wide open and steady for as long as possible. To avoid transients because of vertical drift of the tear film after blinks, the image capture was initiated approximately 2.5 seconds after the third blink. Each subject had their right eye imaged twice to obtain two sets of images/videos. After tear film imaging, tears and meibum samples were collected from the right eye of each subject using collection methods optimized for collecting tear film and meibum OAHFAs, as described previously.^48^^,^^59^^–^^63^

### OCT Imaging

A combined ultra-high-resolution OCT and thickness-dependent fringes interferometer system was used to image the tear film in vivo. This system is capable of capturing images of the tear film with an axial resolution of 1.38 µm. A detailed description of the optical design of this system is available elsewhere.^64^ In brief, the OCT system houses a broadband superluminescent diode (T850-HP; Superlum Diodes, Ltd, Moscow, Russia) with a central wavelength of 840 nm and a full-width half-maximum of 175 nm as an illumination source, whereas the thickness-dependent fringe system consists of a Quartz Tungsten-Halogen Lamp (QTH10; Thorlabs, Newton, NJ, USA). During imaging, the OCT system captures eight horizontal line scans (each comprising of 128 A-lines), evenly spaced along a superior-inferior axis, within a 3 mm–diameter central zone. The acquisition time of each OCT C-scan is approximately 30 ms at a rate of 32 frames per second.

### Mass Spectrometry

A semitargeted direct infusion electrospray MS and MS/MS^ALL^ in the negative ion mode was performed on a TripleTOF 5600 mass spectrometer (SCIEX, Framingham, MA, USA) to assess the abundance of OAHFAs in tear and meibum samples. This analytical approach has been previously validated to provide reliable assessments of lipids in small volumes of the tear film and quantify concentrations of polar lipids, including OAHFAs.^48^^,^^61^ The extraction and mass spectrometry analysis protocols associated with this technique have been described in detail in prior reports.^61^^,^^63^

### Data Analyses

The rates of PCTF thinning were calculated using linear regression fits of PCTF thicknesses derived from the temporal series of OCT images, as described previously.^56^^,^^64^ Data on PCTF thinning rates for each disease classification group are available elsewhere.^56^ For the analysis of MS data, peak intensities of individual OAHFAs identified in meibum and tear samples were normalized by the internal standard intensity to obtain standard-corrected intensity. Data were then imported and analyzed in R^65^ and RStudio.^66^ To avoid issues in downstream analyses, intensities of undetected OAHFAs (zero values) were replaced with a small imputed value (one-fifth of the minimum peak intensity of a particular OAHFA). Data corrected by internal standard were then normalized by the total ion current, mean-centered and subjected to Pareto scaling. For dimensionality reduction, principal component (PC) analyses were performed using “prcomp” function of the “stats” package (v3.6.2) to project the high-dimensional data to a few components that capture most of the variation in the data. Data on tears and meibum OAHFAs were analyzed separately.

The primary outcome of this study was the association between the abundance of OAHFAs and PCTF thinning rate (evaporation). Because some PC scores (weighted average of all features) and PCTF thinning measures were not normally distributed, the associations between PCs (first and second) and PCTF thinning rates were analyzed using Spearman's correlations (ρ). If the association was significant, the absolute loadings of the PCs were ranked, and a list of 10 OAHFAs that had the highest loadings and contributed the most to the PC was derived. Finally, Spearman's correlations were performed to evaluate the association between standard-corrected intensity of these OAHFAs and PCTF thinning rate. Data are expressed as mean ± standard deviation of the mean unless otherwise stated. The value of alpha was set at 0.05 for statistical significance.

## Results

### Baseline Characteristics

Of the 222 subjects consented, 195 were eligible based on classifications conducted using data collected during the clinical examination. Out of these 195 subjects (age 18–84 years; 62.6% female), 178 subjects had both tear film samples and imaging data collected, while 170 subjects had both meibum samples and imaging data collected. The Table summarizes the baseline characteristics of these subjects.

**Table.** Baseline Characteristics of the Study Subjects

	Tears	Meibum
Number of subjects	178	170
Total OAHFAs	78	76
Gender		
Male	65	66
Female	113	104
Age (y), mean (SD)	39.3 (14.2)	39.4 (14.3)
Ethnicity		
Hispanic	1	1
Non-Hispanic	177	169
Race		
Asian	18	19
African American	93	88
Caucasian	65	60
Other	2	3
Volume collected (µL), mean (SD)	0.03 (0.01)	0.03 (0.01)

### Meibum-Derived OAHFAs

**Figure 2.**
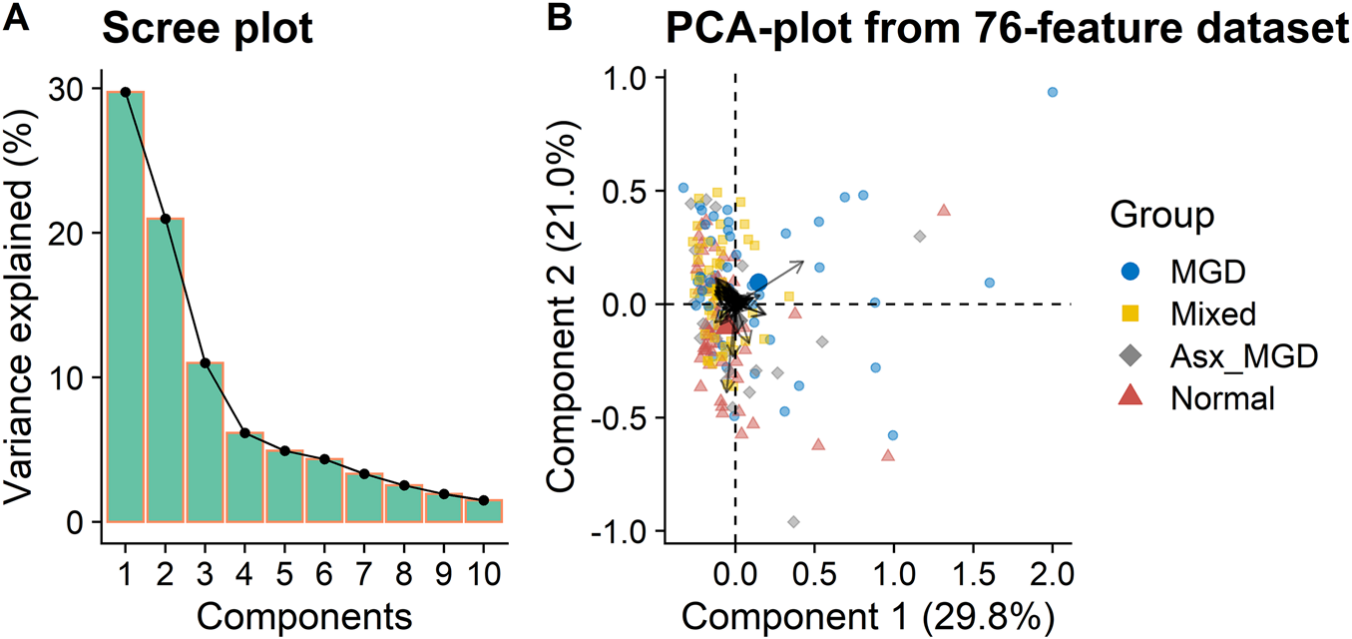
PC analysis of meibum-derived OAHFAs. (**A**) Scree plot showing the proportion of variance explained by the first 10 PCs. (**B**) PC biplot showing sample scores stratified by the four disease classification groups and feature loadings (*black arrows*).

A total of 76 unique OAHFA species were identified in meibum across all subject samples. Figure 2 shows the results of the unsupervised multivariate analyses conducted using the PC method that included all 76 features. As shown in the scree plot (eigenvalues or proportion of variances of PCs) in Figure 2A, the first two PCs captured more than 50% of the variation in the data, with the first PC accounting for 29.8% variance, and the second PC accounting for 21.0% variance. Although most samples projected into a small region forming a narrow herd as illustrated by the PC scores in Figure 2B, a few samples were separated from the herd, indicating some separation among the four classification groups. However, there was little difference among the contributions of the features (black arrows in Fig. 2B). Among the 76 OAHFAs (features), one OAHFA (18:2/16:2) showed a relatively large vector length, suggesting a significantly greater contribution to the PCs than other OAHFAs (features).

**Figure 3.**
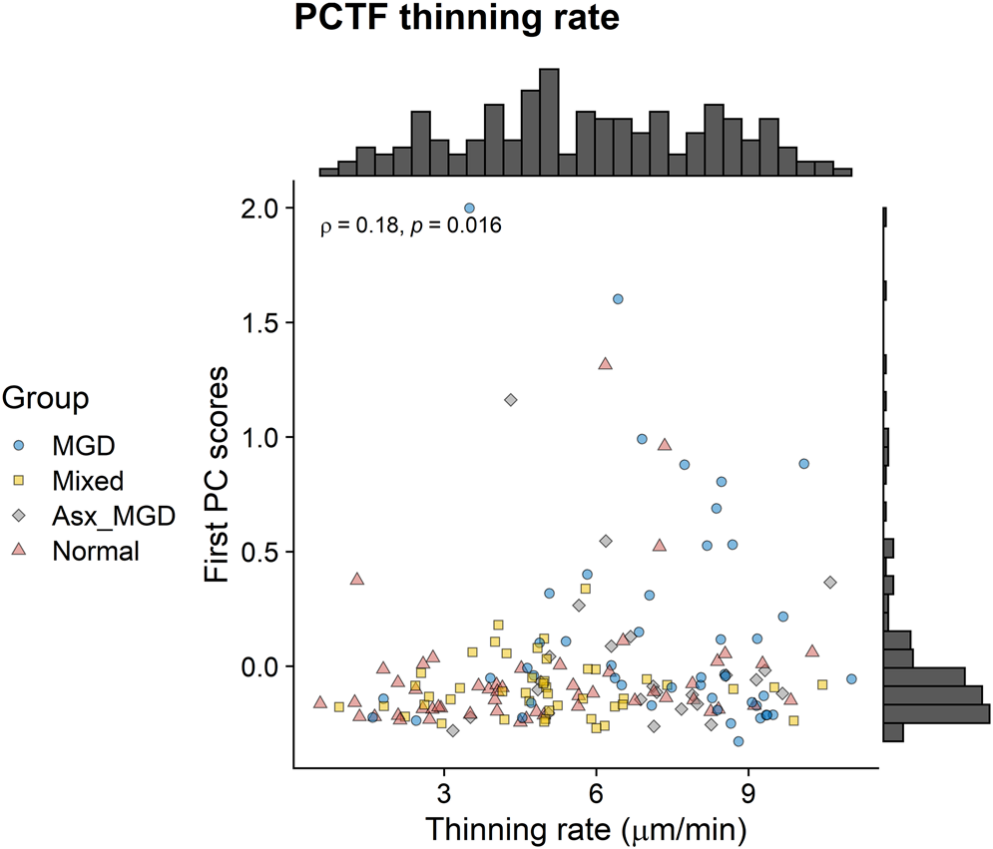
Distributions of the first PC scores of the meibum-derived OAHFAs as a function of PCTF thinning rate. There was a significant positive correlation between the first PC scores and PCTF thinning rate.

To evaluate the association between the PCs and imaging parameters, Spearman's correlation analyses were conducted between the sample scores obtained from PC analyses and PCTF thinning rate (Fig. 3). Although the first PC scores correlated positively with PCTF thinning rate (*ρ* = 0.18, *P* = 0.016), the second PC scores were not associated with PCTF thinning rate (*ρ* = −0.01, *P* = 0.89). In addition, no significant associations were found between the first PC scores and PCTF thickness (*ρ* = 0.10, *P* = 0.18) or average noninvasive keratography break-up time (*ρ* = −0.09, *P* = 0.24).

**Figure 4.**
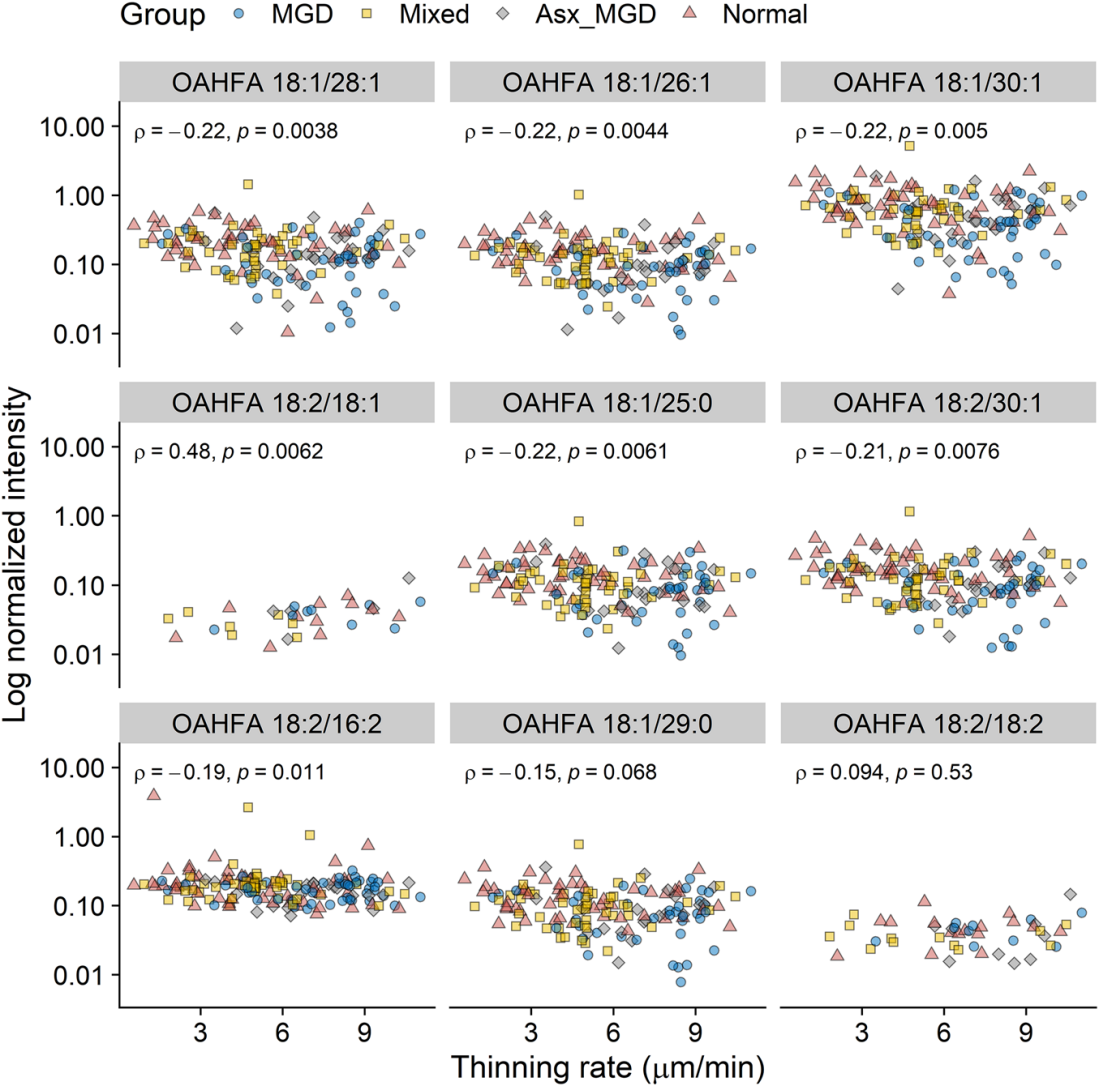
Association between PCTF thinning rate and the abundances of OAHFAs that contributed the most to the first PC. Asx_MGD, asymptomatic MGD.

To identify the most contributing OAHFAs, the absolute values of the first PC loadings of all features were ranked in descending order, and 10 OAHFAs with the highest loadings were selected. Figure 4 shows the correlations between the abundance of these OAHFAs (expressed as standard-corrected intensity) and PCTF thinning rate. One OAHFA (18:0/20:0) was detected in only one sample and therefore omitted from subsequent analysis. Of the other nine OAHFAs, only OAHFA 18:2/18:1 had a positive correlation with PCTF thinning rate (*ρ* = 0.48, *P* = 0.006), whereas the abundance of the other OAHFAs had negative correlations with PCTF thinning rate. These were: OAHFA 18:2/16:2 (*ρ* = −0.19, *P* = 0.01), OAHFA 18:2/30:1 (*ρ* = −0.21, *P* = 0.008), 18:1/28:1 (*ρ* = −0.22, *P* = 0.004), 18:1/30:1 (*ρ* = −0.22, *P* = 0.005), 18:1/25:0 (*ρ* = −0.22, *P* = 0.006), and 18:1/26:1 (*ρ* = −0.22, *P* = 0.006). These results indicate that subjects with greater abundances of these OAHFA in their meibum samples had slower rates of PCTF thinning.

### PCTF-Derived OAHFAs

**Figure 5.**
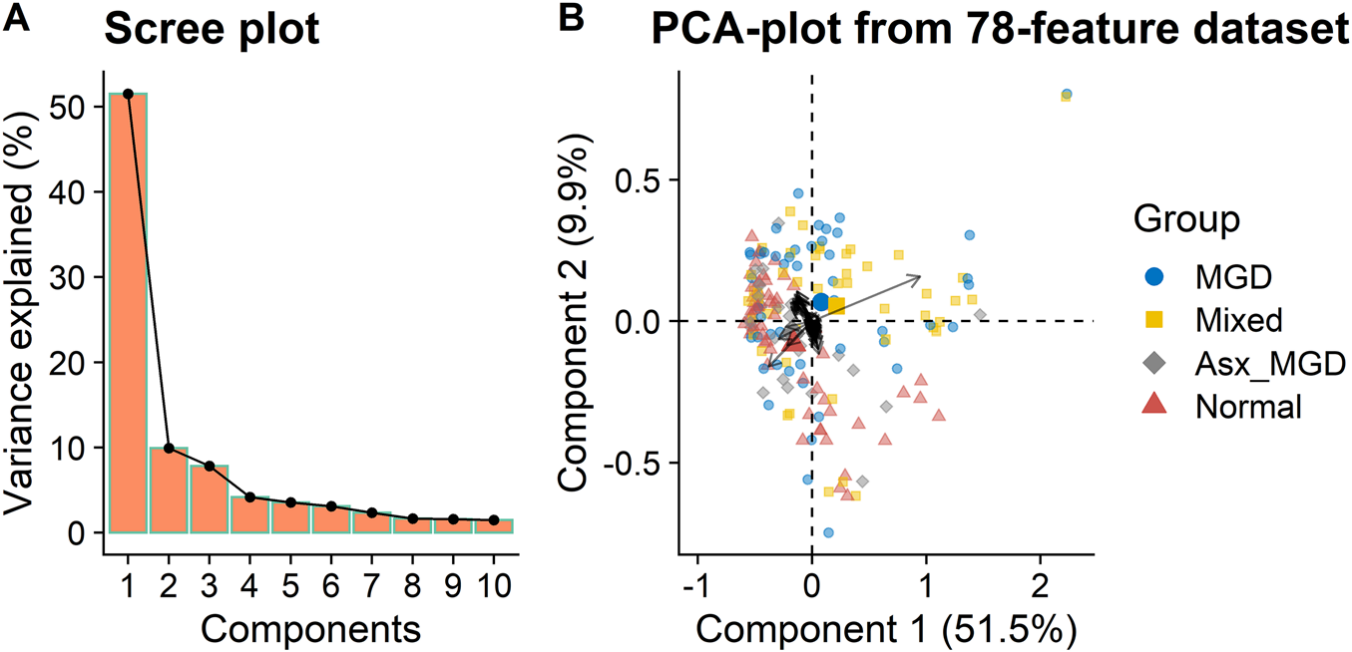
PC analysis of PCTF-derived OAHFAs. (**A**) Scree plot showing the proportion of variance explained by the first 10 PCs. (**B**) PC biplot showing sample scores stratified by the four disease classification groups and feature loadings (*black arrows*).

A total of 78 unique OAHFA species were identified in tear samples across all subjects. Figure 4 illustrates the results of the PC analyses of these 78 features. As shown in the scree plot (Fig. 5A), the first two PCs explained over 60% of the variation in the data, with the first PC accounting for more than 50% of the variance, and the second PC accounting for approximately 10% variance. The proportion of variance explained by the first two PCs was greater for the tear data (61.4%) than the meibum data (50.8%). Most tear samples projected into a small region forming a narrow herd (Fig. 5B). However, there was a slightly greater separation among the groups when compared with the meibum data, and a relatively larger proportion of samples separated from the herd, indicating relatively greater separation among the four disease classification groups. However, the difference among the contributions of the features (black arrows in Fig. 5B) was small. Among the 78 features (OAHFAs), only one feature (OAHFA 18:2/16:2) showed a relatively large vector length (black arrow), suggesting a significantly greater contribution to the PCs than other features.

**Figure 6.**
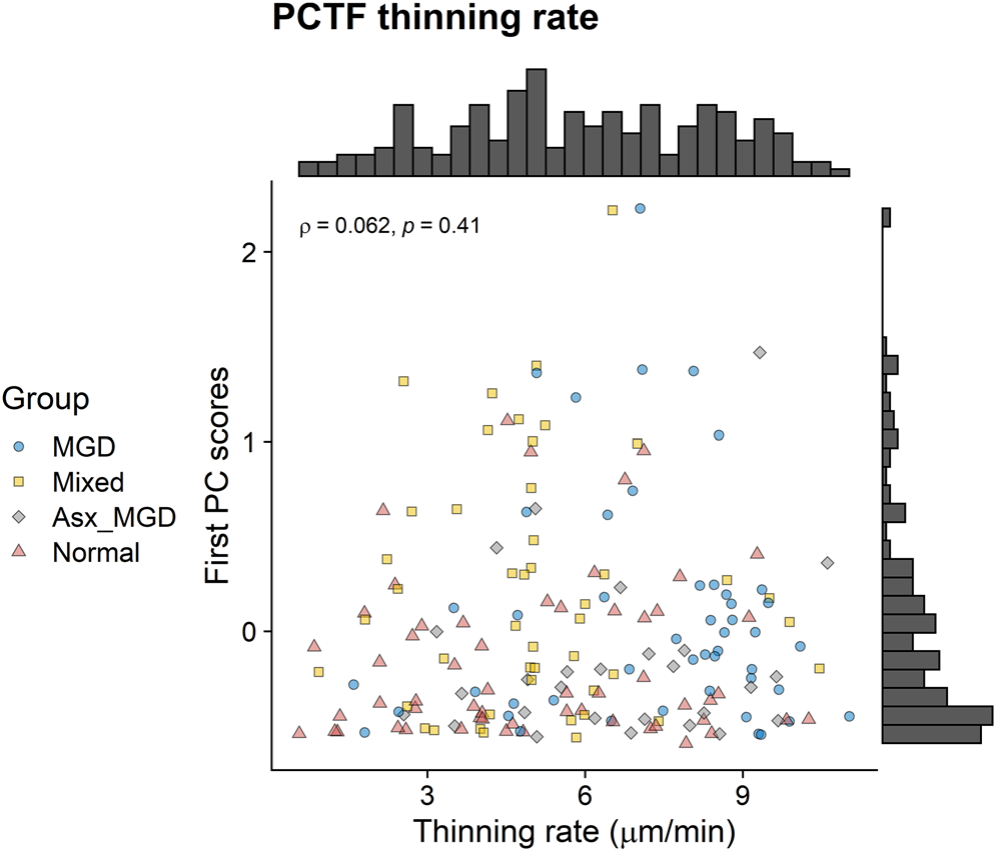
Distributions of the first PC scores of the PCTF-derived OAHFAs as a function of the PCTF thinning rate. There was no association between the first PC scores and the PCTF thinning rate.

Spearman's correlation analyses showed no associations between the first PC scores and PCTF thinning rate (*ρ* = 0.06, *P* = 0.41, Fig. 6). Similarly, the second PC scores also had no associations with the PCTF thinning rate (*ρ* = 0.03, *P* = 0.66). These results suggested that the PCTF-derived OAHFAs were relatively independent of the thinning rate. In addition, no significant associations were found between the first PC scores and PCTF thickness (*ρ* = −0.06, *P* = 0.42) or average non-invasive keratography break-up time (*ρ* = 0.04, *P* = 0.62).

## Discussion

This study set out to investigate the relationship of the amphiphilic polar lipids, OAHFAs, derived from the human tear film and meibum with PCTF thinning (evaporation). The results of the study showed that the abundance of several meibum-derived OAHFAs varied according to PCTF thinning rate. This finding supports the hypothesis that OAHFAs are implicated in the biochemical mechanism underlying evaporation and PCTF thinning and provide evidence that these polar amphiphilic molecules may serve as a candidate molecular biomarker of the PCTF stability in health and disease.

Consistent with the findings from several previous studies,^46^^–^^50^ this study also detected a large number of OAHFAs in the human PCTF (n = 78) and in meibum (n = 76). Several meibum-derived OAHFAs (18:2/18:1, 18:2/16:2, 18:2/30:1, 18:1/28:1, 18:1/30:1, 18:1/25:0, 18:1/26:1) were found to be associated with PCTF thinning, suggesting that these OAHFAs could potentially act as a regulator of PCTF thinning and stability. What biophysical properties distinguish these OAHFAs from the others and make them particularly relevant to PCTF evaporation remain unknown. Most of these OAHFAs were unsaturated with one or more double bonds in the acyl chain length. Therefore the degree of unsaturation could be a critical property of OAHFAs. The absence of double bonds in saturated fatty acids provides a flexible linear shape allowing for greater association with “self.” By contrast, the presence of one or more double bonds in unsaturated fatty acids makes packing with “self” more difficult. This could allow greater opportunity for interdigitation with the non-polar counterparts (“non-self”), providing better TFLL stability and a more effective barrier against PCTF evaporation.^44^^,^^67^^–^^69^ Although most OAHFAs had an inverse association with PCTF thinning rate, suggesting that the abundance of these OAHFAs reduced with an increased rate of PCTF thinning or evaporation, there was a positive relationship between the abundance of OAHFA 18:2/18:1 with PCTF thinning rate. Considering the complex lipid composition of the TFLL,^21^^,^^70^ different OAHFAs may interact differently with the mucoaqueous phase and non-polar lipids and may serve different functions as they relate to tear evaporation.^4^^,^^71^ It is also possible that the ability of the TFLL to retard evaporation depends on a delicate homeostatic balance among several OAHFAs in the TFLL.

An important question remains regarding how these specific OAHFAs regulate the rate of PCTF thinning and evaporation. The TFLL at the lipid-air interface contains a hydrophobic nonpolar lipid sublayer of cholesterol and wax esters connected to the mucoaqueous phase by the amphiphilic polar lipid sublayer, which predominantly consists of OAHFAs and cholesterol-OAHFAs (∼1%–5%), with small amounts of triglycerides, fatty acids, and phospholipids (∼1%).^19^^,^^23^^,^^72^ It has been postulated that this polar lipid sublayer, particularly OAHFAs, facilitates retardation of tear evaporation by stabilizing the nonpolar lipid sublayer.^42^^,^^72^^,^^73^ During eye-opening, the upward extension of the mucoaqueous phase (because of negative pressure in the upper eyelid) and the presence of TFLL lipids on the lower eyelid region creates a surface pressure gradient, which is nullified by the subsequent upward spreading of TFLL on to the mucoaqueous phase.^74^ The latter process requires TFLL to interact with the mucoaqueous phase and likely involves OAHFAs because of their strong polarity as compared with weakly polar cholesterol and wax esters.^43^^–^^45^ There is some evidence that the OAHFAs can spread readily over large regions of mucoaqueous phase,^45^ retard evaporation and inhibit the development of DED,^41^ and exert a direct evaporation-resistant effect, in addition to stabilizing the TFLL.^44^ Mice deficient in fatty acids involved in OAHFA production (ω-hydroxylase Cyp4f39) develop DED and MGD.^43^ They also show a reduction of OAHFAs and their derivatives and accumulation of tears on the lower eyelids indicative of increased surface tension.^43^ These findings suggest that OAHFAs likely act as surfactants to reduce surface tension at the tears/air interface and thus stabilize TFLL to retard tear evaporation and promote PCTF stability.^19^^,^^45^ It has also been shown that the nonpolar sublayers have no contribution to the evaporation-resisting property of the TFLL.^44^^,^^75^ Furthermore, the defects in the TFLL and its coverage across the ocular surface have been reported to primarily drive the rate of tear evaporation.^76^ By stabilizing the TFLL, OAHFAs presumably reduce structural defects in the lipid layer and guard against evaporation. Whereas the antievaporative function of the TFLL may also involve phospholipids, which have been reported in the polar sublayer of the human lipid layer,^20^ the very low concentration of phospholipids in the human tear film (<0.1%^72^) means that they may be unable to interact sufficiently with the nonpolar lipids to stabilize the TFLL.

Compared with the meibum data, the first two PCs explained greater variance in the abundance of tear film-derived OAHFAs, suggesting that the PC technique allowed for significantly better ordination of OAHFAs derived from the PCTF than meibum. It is possible that, unlike meibum, the PCTF contains two distinct sets of OAHFAs: meibomian molecules and oxidized aged molecules. In this regard, the lack of association between tear film-derived OAHFAs and tear film characteristics found in this study is slightly surprising. Tears contain a significantly greater concentration of phospholipids than meibum.^19^ These excess phospholipids are likely derived from sources other than meibum (e.g., cells deposited during blinks) and may interact with meibum-derived OAHFAs in their antievaporative functions. Another contributing factor could be the relatively larger variability in the amount of tear film–derived OAHFAs and tear film imaging measures across the subjects (see Fig. 6). Although a high-resolution untargeted MS method as used in this study has been previously shown to provide reliable characterization and quantification of OAHFAs derived from human meibum and tear film,^48^^,^^61^ this technique yields several candidate lipids requiring a multivariate analytical approach to transform a high-dimensional data to a low-dimensional data. This presents a considerable challenge to investigate the role of specific OAHFAs, particularly if only a few OAHFAs are involved in the mechanism underlying tear evaporation as is evident from the meibum data. Further studies with a targeted approach with a prior chromatographic step may improve our understanding of the role of tear-film–derived OAHFAs in maintaining TFLL and PCTF stability.^47^

In conclusion, this study provides the first evidence of the relationship between OAHFAs and PCTF thinning (evaporation) in a large cohort of healthy and MGD subjects. Results from this study demonstrate that the abundances of several OAHFAs derived from human meibum are associated with PCTF thinning (evaporation); however, OAHFAs derived from the PCTF are independent of the rate of PCTF thinning. The findings of this study are novel and bridge a crucial gap in our understanding of the PCTF homeostasis in health and disease, in addition to providing a basis for further investigations of the mechanistic basis of evaporation-induced PCTF thinning and instability.
